# Corona-Associated Mucormycosis: Case Series Reports and Review of the Literature

**DOI:** 10.3390/jof10050305

**Published:** 2024-04-24

**Authors:** Andreea Fitero, Nicoleta Negrut, Harrie Toms John, Anca Ferician, Paula Marian

**Affiliations:** 1 Department of Psycho-Neuroscience and Recovery, Faculty of Medicine and Pharmacy, University of Oradea, 410073 Oradea, Romania; andreea_gyori@yahoo.com; 2 Doctoral School of Biomedical Sciences, Faculty of Medicine and Pharmacy, University of Oradea, 410087 Oradea, Romania; 3 Department of Ear, Nose and Throat, University Hospitals of Northamptonshire, Cliftonville, Northampton NN1 5BD, UK; tomsharrie@gmail.com; 4 Department of Medical Disciplines, Faculty of Medicine and Pharmacy, University of Oradea, 410073 Oradea, Romania; anca.moza@yahoo.com (A.F.); paula.marian85@gmail.com (P.M.)

**Keywords:** COVID-19, mucormycosis, zygomycosis, black fungus, *Mucorales*

## Abstract

During the COVID-19 pandemic, a significant increase in cases of mucormycosis was observed in COVID-19 patients, especially in India, but not exclusively. The presented cases highlight the heterogeneous nature of mucormycosis, emphasizing the importance of recognizing predisposing factors, such as immunosuppression, due to comorbidities or medication. Diagnosing mucormycosis poses a challenge due to nonspecific clinical manifestations, requiring a multidisciplinary approach for accurate diagnosis. Treatment involves a multi-pronged approach centered around the early initiation of antifungal therapy alongside surgical intervention and the management of underlying conditions, with an emphasis on controlling immunosuppression. Understanding the relationship between COVID-19 and predisposing factors for mucormycosis is fundamental for developing prevention and treatment strategies.

## 1. Introduction

In recent years, the COVID-19 pandemic has affected millions of people worldwide, questioning global health and prompting individuals to reevaluate their lifestyle and hygiene habits. Within this pandemic, a distinct category of individuals was identified as particularly vulnerable, namely those with immunosuppression.

Mucormycosis, also known as ‘zygomycosis’, or ‘black fungus’ is a rare, highly lethal angio-invasive fungal infection caused by a fungus of the *Zygomycetes* class, *Mucorales* order, with species from the genera *Rhizopus* (R) [*R. arrhizus* (*oryzae*), *R. microsporus*], *Mucor* (M) (*M. circinelloides*), *Rhizomucor* (Rh) (*Rh. pusillus*), *Cunninghamella* (C) (*C. bertholletiae*), *Apophysomyces* (A) (*A. variabilis*), *Saksenaea* (S) (*S. vasiformis*), and *Lichtheimia* (L) (*Absidia*) (*L. Corymbifera*, *L. raosa*) the most common. These fungi are commonly found in the environment, and infection rarely occurs in healthy individuals. However, immunocompromised patients are at increased risk of developing mucormycosis [1,2].

Mucormycosis can impact various parts of the body, including the sinuses, lungs, brain, and other organs. The infection often initiates in the sinuses or upper respiratory tract and can rapidly spread to other areas of the body, posing the potential for fatality [2]. The most common forms of mucormycosis are rhino-orbital, cerebral, and sino-pulmonary, with the cutaneous form resulting from spore inoculation due to trauma [3]. Symptoms of the disease may include rhinorrhea, headache, fever, and nasal congestion. Once it reaches the upper respiratory tract, it causes necrosis of the palate, turbinates, and other nasal structures [4]. Mucormycosis leads to thrombosis and death of the affected tissues [5]. The mortality rate of patients with mucormycosis can reach up to 80% [6].

The Mucorales order, besides predominantly affecting immunosuppressed individuals who lack a robust immune arsenal to limit infection, is also responsible for severe forms of the disease. Additionally, these fungi have a high resistance capacity to common antifungals. The correlation of all these factors leads to increased mortality among patients with mucormycosis [7].

The purpose of this case series is to improve the management and outcomes of patients with corona-associated mucormycosis by providing relevant data and information.

This study was approved by the Ethics Committee of the Bihor County Emergency Clinic Hospital, Romania (no 33446 from 7 August 2022), and written informed consent for these data was obtained from each patient at the time of admission to the hospital.

## 2. Case Presentation

### 2.1. Case 1

**Figure 1:**
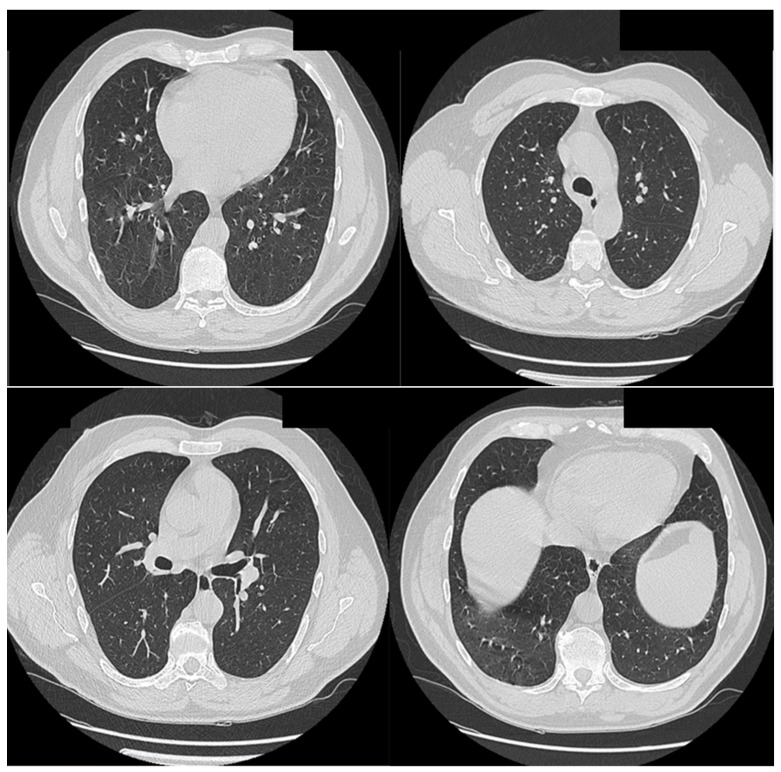
Thoracic CT (scale bar equals 1.25 mm). Fine fibrous band in the right posterior-basal region.

A 50-year-old male patient from a rural area, known to have common B-cell acute lymphoblastic leukemia, was admitted to the Infectious Diseases Department of Bihor County Clinical Hospital, Romania, on 13 August 2022, with a diagnosis of COVID-19 presenting with fever, and received treatment as a result. The patient underwent previous prophylactic antifungal treatment with fluconazole. Chest computed tomography (CT) revealed a fine fibrous band in the right posterior basal region, Figure 1. The paraclinical investigations conducted are presented in Table S1. Treatment was initiated with anti-viral (molnupiravir, for 5 days), corticosteroid (dexamethasone, for 10 days), antibiotics (meropenem, linezolid), for 10 days, platelet transfusion, antipyretics, and antifungal medications (fluconazole) for 14 days.

**Figure 2:**
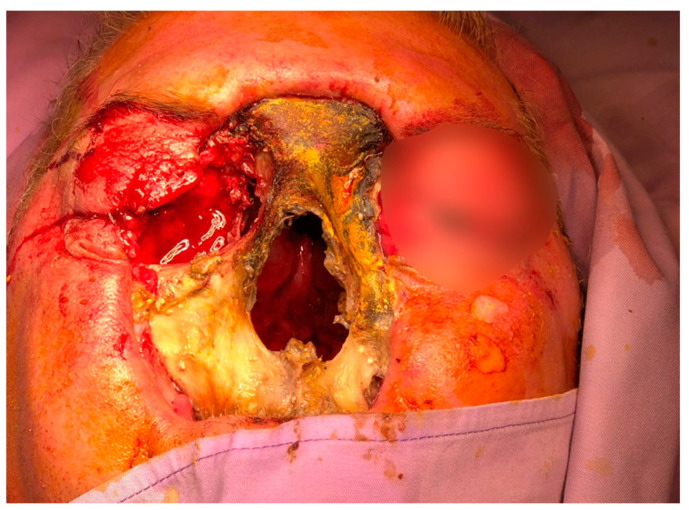
The clinical aspect of the patient. Edema in the upper and lower eyelids with diffuse facial edema and centrofacial vascular necrosis at the level of the nasal pyramid with right orbital extension and involvement of the right maxillary sinus.

**Figure 3:**
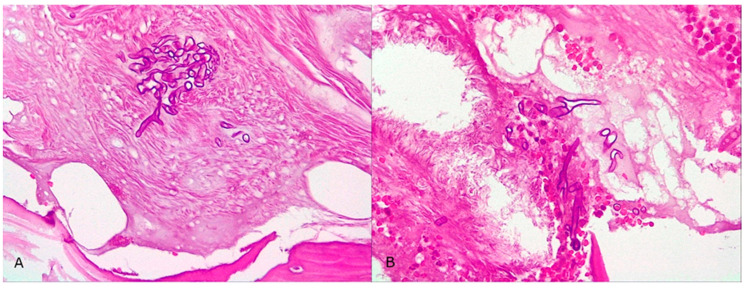
Microscopic examination of the biopsy specimen (400× magnification). (**A**,**B**) Multiple histological sections represented by necrotic-inflammatory material, with numerous bacterial colonies and fungal filaments, some branching at a 90-degree angle, consistent with mucormycosis.

**Figure 4:**
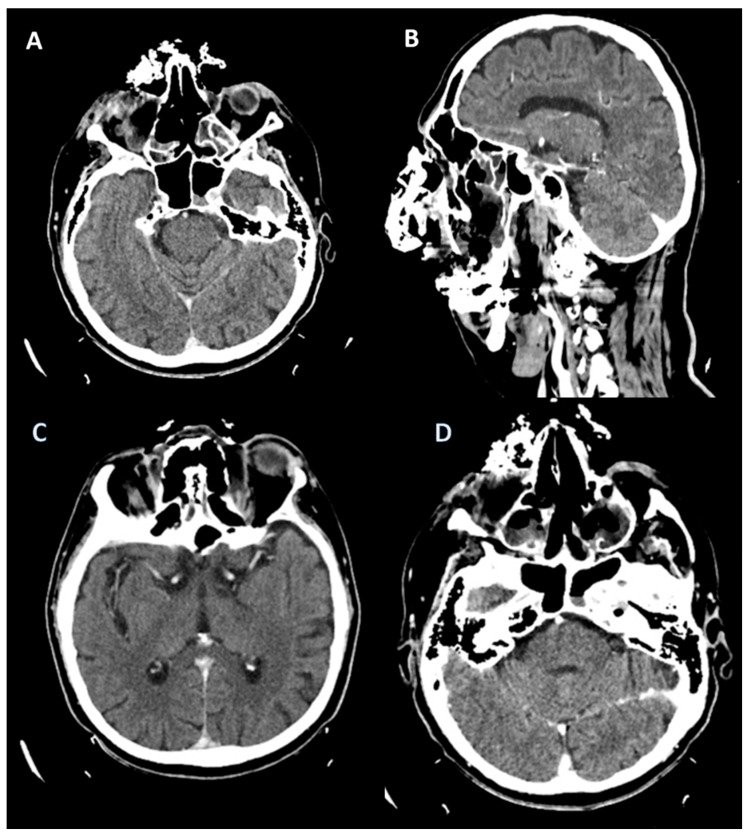
Cranial CT (scale bar equals 1.25 mm). (**A**,**B**) The image of the coronal computed tomography shows thickening of the mucosa and homogeneously walled-off collections with air inclusions, localized in the left sphenoidal sinus, bilateral frontal maxillary sinuses, and ethmoidal cells, post-necrotic bone structure changes with the interruption of the bone cortex, and multiple osteolytic lesions in the bilateral fronto–ethmoido–maxillary regions, as well as in the nasal septum and nasal bones. (**C**,**D**) Right eyeball with reduced dimensions and altered structure with a shrunken appearance.

Subsequently, on 30 August 2022, he developed edema in the upper and lower eyelids, diffuse facial edema with Celsian signs, and associated centrofacial vascular necrosis at the level of the nasal pyramid with right orbital extension and the involvement of the right maxillary sinus, accompanied by headache, leading to admission to the Ear Nose Throat (ENT) department, Figure 2. A macroscopic anatomopathological examination revealed multiple necrotic fragments and a microscopic examination showed multiple histological sections represented by necrotic-inflammatory material, with numerous bacterial colonies and fungal filaments, and some branching at a 90-degree angle, consistent with mucormycosis, Figure 3. Cranial CT showed massive destructive infectious processes involving the maxillary sinuses, posterior ethmoid cells, and orbits with involvement of the right eye globes, possibly of mixed fungal and bacterial etiology, Figure 4. Surgical treatment was instituted by performing a total necrectomy of the centrofacial necrotic focus along with antibiotics (amoxicillin-sulbactam, cefoperazone) for 10 days and antifungal therapy with posaconazole (for 9 weeks) due to myelosuppression. Amphotericin B and isavuconazole were not available at that time in the hospital. Postoperative recovery was satisfactory, and subsequently, he was transferred to the hematology service for further specialized treatment.

### 2.2. Case 2

**Figure 5:**
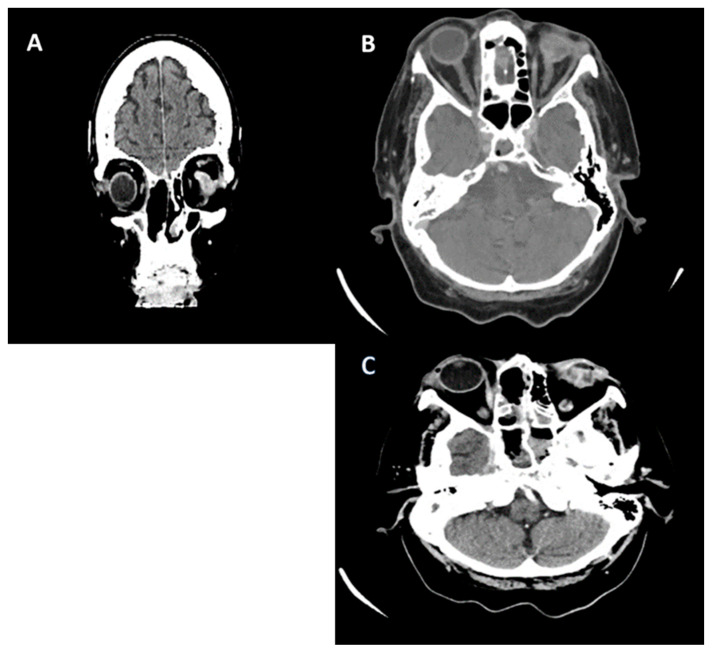
Cranial CT. (**A**) (scale bar equals 1.25 mm). Left ocular globe with loss of sphericity, reduced dimensions, and heterogeneous hyperdense appearance of the vitreous body with subcorneal and subscleral air infiltrates. (**B**,**C**) Thickening of the mucosa of the right maxillary sinus with lysis of the postero-superior wall, as well as the posterior right ethmoid with a collection showing fluid and parafluid densities at that level, with an iodophilic wall communicating with another collection with the same characteristics in the projection of the right sphenoid, which presents lysis of the anterior wall, lysis of the anterior ethmoid cells, and the nasal bones on the right side, as well as a collection in the right frontal sinus.

**Figure 6:**
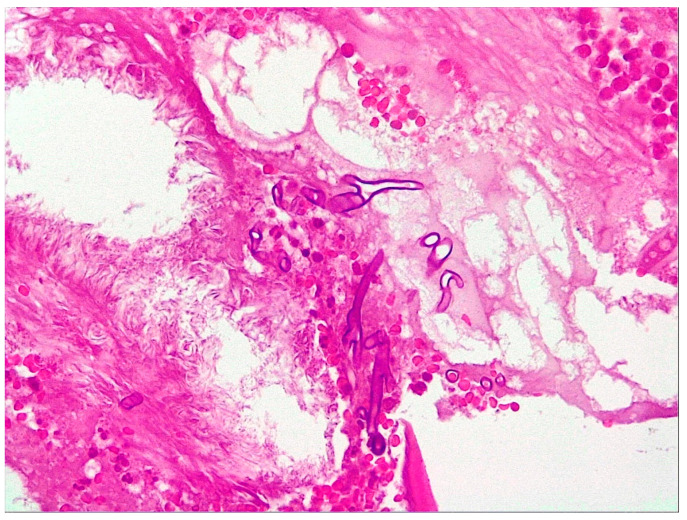
Microscopic examination of the biopsy specimen (400× magnification). Fragments with necrosis, inflammation, and hyphae in the form of thick ribbons measuring 10–20 microns and some elongated.

An 84-year-old female patient from an urban environment, known to have insulin-dependent type 2 diabetes mellitus, was admitted in August 2023 to the Ear, Nose and Throat (ENT) Department of the Bihor County Clinical Hospital, Romania, presenting with headache and swelling of the right eyelid. It is noteworthy that three weeks ago, the patient had a SARS-CoV-2 infection, which was managed at home (including moxifloxacin and dexamethasone, both for 10 days). On physical examination, she presented with necrosis of the mucosa and inferior nasal turbinate on the right side, with occasionally bloody and foul-smelling mucopurulent secretions, purulent endophthalmitis of the right eye, right eye blindness, and onychomycosis. A cranial CT scan revealed left orbital cellulitis with endophthalmitis, right pansinusitis with frontal, sphenoidal, and posterior ethmoidal collections, and adjacent bone lysis, Figure 5. Macroscopic histopathological examination revealed a black, foul-smelling crust lining the pituitary mucosa. The microscopic examination showed fragments with necrosis, inflammation, and hyphae in the form of thick ribbons measuring 10–20 microns, some of which were elongated. The microscopic appearance is suggestive of mucormycosis, Figure 6. Surgical treatment was initiated with the excision of necrotic tissues, antifungal therapy (posaconazole), hemostatics, and insulin. At the time of treatment, amphotericin B and isavuconazole were unavailable in the hospital. Laboratory tests showed elevated transaminases, prompting the addition of hepatoprotective agents, Table S2. The subsequent clinical course was favorable with posaconazole (for 6 weeks).

### 2.3. Case 3

**Figure 7:**
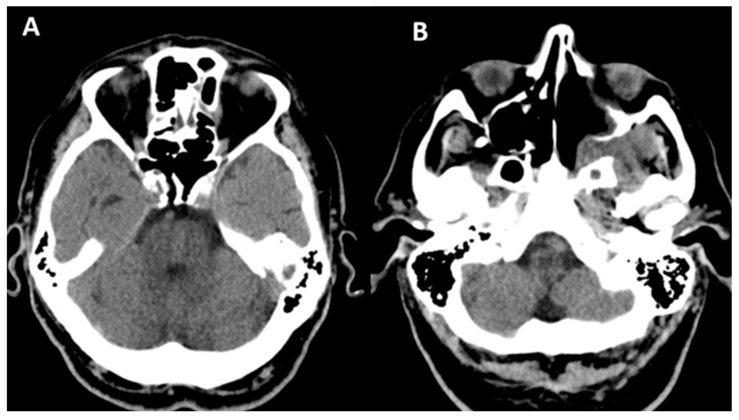
Cranial CT. (**A**,**B**) (scale bar equals 1.25 mm). Parafluid collection at the level of the left maxilla with osteolysis of the anterior, medial, and posterior walls; a similar aspect was observed at the level of the left sphenoidal sinus in its basal portion with osteolysis of the antero-inferior wall. Minimal thickening of the mucosa of the left frontal sinus and left ethmoidal cells, with the absence of the left nasal turbinate.

**Figure 8:**
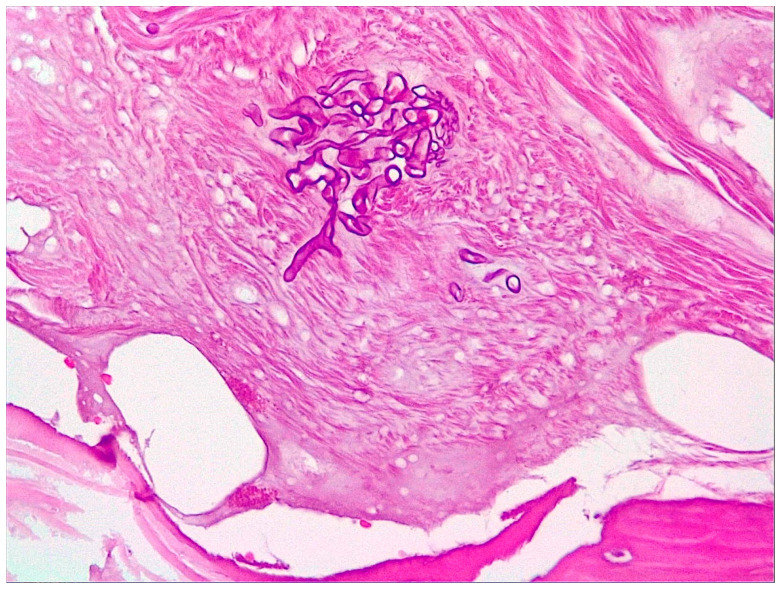
Microscopic examination of the biopsy specimen (400× magnification). Non-septate hyphae angled at 90 degrees, along with areas of chronic inflammation and bacterial colonies.

A 63-year-old male patient from a rural area was admitted to the ENT Department of Bihor County Clinical Hospital, Romania, in January 2022, complaining of headache, swelling of the left half of the face, and mucopurulent rhinorrhea. On physical examination, extensive necrosis of the left nasal cavity mucosa with black crusts and yellow-blackish secretions and a complete absence of the middle nasal turbinate were noted. The patient had a history of SARS-CoV-2 infection two months before onset (untreated with corticotherapy or antibiotics). A cranial CT scan revealed left pansinusitis with an extension of the inflammatory process to the left pterygoid musculature, Figure 7. Microscopic histopathological examination revealed non-septate hyphae angled at 90 degrees, along with areas of chronic inflammation and bacterial colonies, Figure 8. Periodic Acid-Schiff staining highlighted these hyphae, which were interpreted as mucormycosis. Surgical treatment was initiated with the debridement of necrotic areas, antifungal therapy (amphotericin B for 3 weeks), antibiotics (vancomycin, ciprofloxacin, both for 2 weeks), and analgesics, with favorable progress. On the 8th day of treatment, the patient developed acute renal failure (creatinine 4.98 mg/dL), prompting the discontinuation of vancomycin therapy to reduce nephrotoxicity, with subsequent favorable evolution and creatinine decreasing to 1.56 mg/dL, Table S3.

## 3. Discussion

One perplexing complication observed during the COVID-19 pandemic is the surge in life-threatening mucormycosis infections with COVID-19, having a psychological impact on patients [8]. Understanding the intricate molecular interplay between COVID-19 and susceptibility factors that set the stage for mucormycosis can inform prevention and treatment strategies against this disfiguring and invasive fungal disease [9].

The pathogenesis of mucormycosis involves the inhalation or direct inoculation of fungal spores, followed by germination and angioinvasion within susceptible tissue [10]. Several key factors allow the fungus to successfully attach to and invade host tissues. The primary attachment receptors used by *Rhizopus* are host cell glucose regulators, including the GRP78 receptor [11]. *Rhizopus* spores release cotH proteins that bind to these glucose regulators with high affinity, facilitating cell penetration following spore attachment [11]. Individuals with uncontrolled hyperglycemia, such as in diabetic ketoacidosis or other severe hyperglycemic states, have increased the expression of glucose regulators on the cell surface. They are, therefore, at significantly increased risk for mucormycosis due to ample binding receptors, as the fungus preferentially invades tissues with the highest receptor availability [11,12].

In addition to attachment receptor availability, local tissue factors create an environment that promotes fungal growth and invasion. Tissues with high levels of available serum iron, especially transferrin-bound iron, facilitate the growth of *Rhizopus* [11]. Diabetic ketoacidosis creates low pH environments and elevates free, unbound iron levels through their release from transferrin. Furthermore, diabetic microvascular complications lead to local tissue ischemia and necrosis. The fungus thrives in acidic environments with freely available iron and poorly perfused tissues with lower oxygen tension [13].

The immune status of tissues also impacts the pathogenesis of mucormycosis. The tissue’s immunity influences the pathogenesis of mucormycosis. In leukemia, macrophage activity is inhibited because of corticosteroid therapy, and the number of neutrophils can be directly affected; both events lead to the decreased destruction of swollen spores and hyphae of *Mucorales*. Furthermore, the host’s immune responses are compromised due to the suppression of T lymphocyte induction, interferon-gamma production, and phagocyte-mediated killing. Chemotherapy, used in oncology patients, induces immunosuppression, leading to secondary infections with various microorganisms. Immunosuppression in these cases can be caused by mechanisms of direct cytotoxicity, the alteration of development, or the functionality of immune system cells. Patients receiving deferoxamine therapy for iron or aluminum overload are at increased risk as deferoxamine chelates transferrin-bound iron, effectively releasing free iron for use by *Rhizopus* [14]. 

The link between COVID-19 and mucormycosis is complex and multifactorial. The SARS-CoV-2 virus uses the angiotensin-converting enzyme-2 (ACE-2) receptor and transmembrane serine protease (TMPRSS2) for host cell entry [15]. Lung alveolar cells with high ACE2 expression are preferred sites of infection and replication. SARS-CoV-2 proteins antagonize innate immune responses, delaying the production of pro-inflammatory cytokines like interferons, tumor necrosis factor α, interleukins (IL)-6, and IL-1β [16]. However, dysregulated inflammation eventually ensues, with a hyperinflammatory “cytokine storm” causing tissue damage while failing to clear the virus [16]. This results in diffuse alveolar damage in the lungs, destroying physical barriers to infection while flooding alveoli with nutrients that foster microbial growth [17]. Mucorales spores lying dormant in lung alveoli or sinuses can later penetrate and infect susceptible tissue [17]. 

Invasive pulmonary aspergillosis demonstrates similarities in its pathogenesis—the cytokine storm, tissue necrosis, and immune cell apoptosis in COVID-19 create an environment that enables normally harmless airborne *Aspergillus* spores to become pathogenic and invasive [18]. Analogously, in mucormycosis, the pro-inflammatory reaction helps breach physical barriers while simultaneously dampening critical immune responses against the fungi [19].

While suppressing early innate responses, SARS-CoV-2 also cleverly evades adaptive immunity. One mechanism involves the nucleocapsid protein inhibition of the cluster of differentiation (CD) 8+ T-cell activation, resulting in reduced anti-viral cytotoxic T-lymphocyte responses [20]. SARS-CoV-2 infection also causes pronounced lymphopenia and the apoptosis of CD4+ and CD8+ T cells, limiting antigen presentation and B-cells, which are critical for antibody production [21]. The loss of lymphocytes combined with overactive myeloid cell responses promotes excessive inflammation while weakening specific anti-viral and antifungal defenses. This transitory immunocompromised state enables normally harmless *Mucorales* that present ubiquitously in nature to shift to aggressive pathogens no longer constrained by functional immune surveillance. 

Similar to other invasive fungi like *Aspergillus*, *Mucorales* appear to have evolved specialized mechanisms for replicating inside phagocytic cells, thereby evading macrophages and neutrophils whose phagocytic action normally limits fungal growth [22]. The fungus *Rhizopus arrhizus* can replicate within macrophages, inducing cell death and disseminating the infection. 

Several molecules can lead to a decrease in the body’s defense system. These include steroid preparations and antibiotics. In severe cases of SARS-CoV-2 with respiratory failure, dexamethasone has been the first line of treatment, proving effective in reducing mortality and leading to an exponential increase in steroid use [23]. Increased steroid consumption has led to a rise in cases of opportunistic infections associated with SARS-CoV-2, such as mucormycosis, aspergillosis, and candidiasis [23]. In healthy hosts, neutrophil recruitment and phagolysosome acidification kill fungal spores. However, in COVID-19 patients, corticosteroids like dexamethasone that are given to treat inflammation may render neutrophils dysfunctional against *Mucorales*. Fungal spread ensures that they are unchecked by either arm of enfeebled immunity [24]. In a study conducted by Singh A.K. et al. on 101 cases of corona-associated mucormycosis reported in the medical literature, 76.3% of patients used corticosteroids [25]. Before the pandemic, global cases of mucormycosis ranged from 0.005 to 1.7 per million people, while in India, the incidence was 70 times higher than the global average, with over 200,000 cases annually, accounting for 24% of all fungal infections [26,27]. During the COVID-19 pandemic, India reported 41,000 cases of mucormycosis associated with COVID-19 by the end of June 2021, resulting in 3200 deaths [28]. 

Microbiota impacts the immune system, and secondary dysbiosis due to antibiotic consumption can influence the body’s immune response. Microbial communities within various microbiotas are interdependent, so the destruction of one can indirectly lead to the depletion of others. Dysbiosis occurring in this context is responsible for the decreased development of intestinal lymphoid tissues and decreased levels of serum immunoglobulins of type G, interferon γ, and interleukins (ILs) involved in promoting the immune system (IL-17, IL-1, IL-18, and IL-36) [29,30].

Additionally, patients with a severe form of SARS-CoV-2 infection required admission to intensive care units and underwent various invasive medical procedures, such as intubation and mechanical ventilation. These procedures can increase the risk of developing nosocomial infections, including mucormycosis, especially if there is inadequate hygiene or improper sterilization of medical equipment. Furthermore, the SARS-CoV-2 virus can affect the body’s immune system and damage mucosal barriers, facilitating the entry and spread of other pathogens, including *Mucorales* fungi. 

The correlation of all these factors leads to increased mortality among patients with mucormycosis [31]. Even patients with intact immune systems often cannot prevent fungal dissemination due to the organism’s rapid growth rate.

The most common form of mucormycosis in immunocompromised patients is rhino-orbito-cerebral [32], as observed in the presentations above. Each case underscores the heterogeneous nature of mucormycosis presentations and the importance of recognizing predisposing factors [33]. Case 1 illustrates the increased susceptibility of immunocompromised individuals, such as those with hematologic malignancies, to mucormycosis, particularly in the context of COVID-19, corticosteroids, and antibiotics therapy. Case 2 highlights the association between mucormycosis and poorly controlled diabetes mellitus, which is compounded by the immunosuppressive effects of SARS-CoV-2 infection, antibiotics, probiotics, and corticosteroid therapy. A link has been established between mucormycosis and uncontrolled diabetes [34,35]. Diabetes mellitus has been the most common predisposing factor (73.4%) for mucormycosis in patients with SARS-CoV-2 [36,37,38]. Case 3 emphasizes the propensity for mucormycosis to affect individuals with a history of SARS-CoV-2 infection, irrespective of their immune status, suggesting a potential link between viral respiratory illnesses and fungal superinfections. In this case, the consumption of steroid preparations and antibiotics is not present. Even though the patient comes from a rural environment, with the possibility of colonization with *Mucorales*, albeit in very rare cases, there exists the fact that it has become clinically evident that immunosuppression is secondary to COVID-19 infection; this idea should be reinforced. Thus, the question arises whether SARS-CoV-2 infection alone does not predispose these patients to mucormycosis [39]. All three cases of corona-associated mucormycosis were presented, regardless of previous immune status, with immunosuppression generated by SARS-CoV-2 infection, prior corticosteroid, and antibiotic therapy.

Diagnosing mucormycosis poses challenges in differential diagnosis, especially for inexperienced medical personnel, due to its nonspecific clinical presentation and the lack of specificity in routine paraclinical tests [40]. Imaging studies are the ones that raise suspicion of mucormycosis. Histopathological examination remains the gold standard for confirming the diagnosis by identifying non-septate hyphae. Etiological diagnosis requires the collaboration of a multidisciplinary team, starting from clinical and imaging suspicion with histopathological confirmation in tissue biopsy samples.

The treatment for coronavirus-associated mucormycosis involves antifungal therapy, surgical intervention, and the management of underlying medical conditions, among which immunosuppression is the most important. Etiological therapy is imperative to initiate as soon as possible in all cases of illness, with liposomal amphotericin B as the first line of medication. Alternatively, posaconazole and isavuconazole can be used [41].

Surgical intervention with clean margins is necessary for the removal of affected tissues and the reduction in fungal burden, thereby facilitating antifungal therapy and improving therapeutic outcomes. Surgical intervention was performed in all three cases presented. Studies support the idea that medical–surgical therapy increases the survival rate of these patients [40].

Managing immunosuppression is essential for therapeutic success and preventing the recurrence of mucormycosis. For these patients, discontinuing immunosuppressive therapies and controlling glycemia increases the chances of an immune response against fungal infection.

Cases of mucormycosis have been extremely rare in the Romania region. Before the COVID-19 pandemic, only seven cases had been diagnosed in our country [42]. During the pandemic period, four more cases were reported from 2021 to 2023 [43,44,45,46]. The three cases reported by us, along with those reported so far in the region of Romania since the onset of the COVID-19 pandemic, equal the total number of cases reported in the same region of Europe before the pandemic. The increase in the number of reported cases in such a short period can be considered alarming for the region of Romania.

## 4. Conclusions

The three cases of coronavirus-associated mucormycosis all exhibited immunosuppression induced by SARS-CoV-2 infection, irrespective of their previous immune status. In two cases, this immunosuppression was exacerbated by prior corticosteroid and antibiotic therapy. These observations highlight the potential susceptibility of COVID-19 patients to mucormycosis, especially when coupled with additional immunosuppressive treatments. The increasing trend in reporting cases of Coronavirus-associated mucormycosis in Romania following the declaration of the COVID-19 pandemic is alarming for this geographical area. Vigilant monitoring and collaborative management are imperative for the timely detection and successful treatment of this severe fungal infection in COVID-19 cases.

## Data Availability

Data are contained within the article and Supplementary Materials.

## Supplementary Materials

The following supporting information can be downloaded at: https://www.mdpi.com/article/10.3390/jof10050305/s1, Tables S1–S3. Laboratory analyses.
