# Supplementary material for: Corona-Associated Mucormycosis: Case Series Reports and Review of the Literature
Source: J Fungi (Basel). 2024 Apr 24;10(5):305. doi: 10.3390/jof10050305 (PMC11122562; doi:10.3390/jof10050305)
Supplement: Supplementary file 1 [file jof-10-00305-s001.zip › Table S2.pdf]

Table S2. Laboratory analyses.

Parameter	*Value*	*Reference Range*
WBC, cells/mm³	13.5 x10³*	4.00-10.00 x10³
Neutrophils, cells/mm³	10.2 x10³*	2.4 - 6.5 x10³
Lymphocytes, cells/mm³	2.07 x10³	1.00 - 4.00 x10³
RBC, cells/mm³	4.40 x 10⁶	3.80 – 5.20 x 10⁶
Hemoglobin, g/dL	13.2	11.7 - 16.1
MCV, fl	95.3 fl	80.0 – 100.0
MCH, pg	30.1	27.0 – 34.0
PLT, cells/mm³	288 x 10³	150 – 450 x 10³
Fibrinogen, mg/dL	388	200 – 393
C-reactive protein, mg/L	8.10*	0 - 5.0
Procalcitonin, ng/mL	0.02	0.00 – 0.05
Prothrombin time, percentage	105 %	70-140
D-dimer, ng/mL	109	0 – 198
Glycemia, mg/dL	200*	82-115
Ferritin, ng/mL	143	13 – 150
ALAT, U/L	70*	0 - 55
ASAT, U/L	40*	5 - 35
Urea, mg/dL	21.00*	9.8 – 20.1
Creatinine, mg/dL	1.01	0.57 – 1.11
Blood cultures	negative	negative
Nasal secretion culture	negative	negative

*WBC – white blood cells; RBC – red blood cells; MCV - mean corpuscular volume; PLT – platelets; ALAT - alanine aminotransferase; ASAT - aspartate aminotransferase; Ag – antigen, * – pathological values.*
