# Supplementary material for: Corona-Associated Mucormycosis: Case Series Reports and Review of the Literature
Source: J Fungi (Basel). 2024 Apr 24;10(5):305. doi: 10.3390/jof10050305 (PMC11122562; doi:10.3390/jof10050305)
Supplement: Supplementary file 1 [file jof-10-00305-s001.zip › Table S3.pdf]

Table S3. Laboratory analyses.

Parameter	*Value*	*Reference Range*
WBC, cells/mm³	9.72 x10³	4.00-10.00 x10³
Neutrophils, cells/mm³	6.49 x10³	2.4 - 6.5 x10³
Lymphocytes, cells/mm³	2.50 x10³	1.00 - 4.00 x10³
RBC, cells/mm³	4.94x 10⁶	3.80 – 5.20 x 10⁶
Hemoglobin, g/dL	13.7	11.7 - 16.1
MCV, fl	85.1	80.0 – 100.0
MCH, pg	27.6	27.0 – 34.0
PLT, cells/mm³	273 x 10³	150 – 450 x 10³
Fibrinogen, mg/dL	651*	200 – 393
C-reactive protein, mg/L	27.40*	0 - 5.0
Procalcitonin, ng/mL	0.80*	0.00 – 0.05
Prothrombin time, percentage	80 %	70-140
D-dimer, ng/mL	235*	0 – 198
Glycemie, mg/dL	100*	82-115
Ferritin, ng/mL	888.18*	13 – 150
ALAT, U/L	6	0 - 55
ASAT, U/L	11	5 - 35
Urea mg/dL	159*	9.8 – 20.1
Creatinine mg/dL	4.98*	0.57 – 1.11
Blood cultures	negative	negative
Nasal secretion culture	Echerichia coli pozitive* Staphylococcus aureus MRSA pozitive*	negative

*WBC – white blood cells; RBC – red blood cells; MCV - mean corpuscular volume; PLT – platelets; ALAT - alanine aminotransferase; ASAT - aspartate aminotransferase; Ag – antigen, * – pathological values.*
